# Real-World Systemic Treatment Patterns after Atezolizumab and Bevacizumab in Patients with Hepatocellular Carcinoma in the United States

**DOI:** 10.3390/cancers15235532

**Published:** 2023-11-22

**Authors:** Amit G. Singal, Kirhan Özgürdal, Xiaozhou Fan, Zdravko Vassilev, Xiaoyun Pan, Jasjit K. Multani, Chi-Chang Chen, Zifan Zhou, Jing He, Federica Pisa

**Affiliations:** 1 Department of Internal Medicine, University of Texas Southwestern Medical Center, Dallas, TX 75390, USA; 2 Medical Affairs Oncology, Bayer Consumer Care AG, 4052 Basel, Switzerland; 3 Oncology RWE, Bayer HealthCare Pharmaceuticals Inc., Whippany, NJ 07981, USA; 4 Global Outcomes Research Department, Bayer HealthCare Pharmaceuticals Inc., Whippany, NJ 07981, USA; 5 Health Economics and Outcomes Research, Real World Evidence Solutions, IQVIA US, Falls Church, VA 22042, USA; 6 Health Economics and Outcomes Research, Real World Evidence Solutions, IQVIA US, Wayne, PA 19087, USA; 7 Advanced Analytics, IQVIA US, Plymouth Meeting, PA 19462, USA; 8 Real World Evidence Oncology, Bayer AG, 13342 Berlin, Germany; federica.pisa@bayer.com

**Keywords:** liver cancer, treatment patterns, immunotherapy, targeted therapy, chemotherapy

## Abstract

**Simple Summary:**

Atezolizumab plus bevacizumab (atezo + bev) is a preferred front-line treatment for unresectable hepatocellular carcinoma (HCC). However, there is limited real-world evidence regarding the use of atezo + bev and subsequent HCC therapies in clinical practice. This retrospective cohort study aimed to characterize the time to discontinuation of atezo + bev, sequencing of systemic therapy after atezo + bev, and time to next treatment. We identified 825 adults with HCC who were initiated on atezo + bev between June 2020 and June 2022. During a median follow-up of 15.3 months, most patients (72%) discontinued atezo + bev, with a median time to discontinuation of 3.5 months. Less than one in five (19%) received subsequent therapies (median time to subsequent treatment of 5.4 months); the most common subsequent agents were lenvatinib (6%), cabozantinib (4%), and nivolumab (4%). Further research is needed to identify those most likely to benefit from atezo + bev and evaluate optimal sequential HCC therapies to maximize overall survival.

**Abstract:**

Real-world (RW) evidence is needed to evaluate atezolizumab plus bevacizumab (atezo + bev) utilization for hepatocellular carcinoma (HCC) in clinical practice. This retrospective cohort study used administrative claims databases to evaluate treatment patterns in individuals with HCC ≥18 years of age who were initiated on atezo + bev between June 2020 and June 2022. The endpoints of this study were the proportion of individuals who discontinued atezo + bev and received subsequent systemic therapies, time to discontinuation (TTD), and time to next treatment. Overall, 825 individuals were eligible (median age 67 years; 80% male). Over a median follow-up of 15.3 months, most (72%) discontinued atezo + bev, with a median TTD of 3.5 months. A minority (19%) received subsequent therapies, with the most common second-line agents being lenvatinib (6%), cabozantinib (4%), and nivolumab (4%). The median time from index to next treatment post-atezo + bev was 5.4 months. Further research is needed to identify the patients who are most likely to benefit from atezo + bev as well as later-line HCC therapies to optimize overall survival.

## 1. Introduction

Hepatocellular carcinoma (HCC) is the most common primary liver malignancy, accounting for 90% of cases, and is the fourth leading cause of cancer-related death globally [1,2,3]. Owing to it often being diagnosed at an advanced stage, around 70–80% of individuals with HCC are ineligible for curative treatments [4,5,6]. The median overall survival for patients with HCC remains poor, with 5-year survival rates of approximately 20%, including 13% for regionally advanced disease and 3% for distant metastatic disease [6,7,8].

Sorafenib, a multikinase inhibitor that demonstrated survival gains in patients with advanced HCC [9,10,11,12], was the first US Food and Drug Administration (FDA)-approved treatment for unresectable HCC and has been the standard of care in the front-line setting since 2007. Starting in 2017, several systemic therapies, including multikinase inhibitors and monoclonal antibodies, were approved for second line use after sorafenib [13,14,15,16]. In 2020, a new standard of care in front-line treatment, atezolizumab plus bevacizumab (atezo + bev) [17], was approved by the FDA for the treatment of unresectable HCC, following the results of the global, open-label, phase 3 IMbrave150 Trial [18]. Tremelimumab, in combination with durvalumab, recently gained FDA approval for the same indication based on positive results from the phase 3 HIMALAYA trial [19].

Updated findings from the IMbrave150 randomized controlled trial (RCT) for patients who were initiated on atezo + bev (*n* = 336) over a median follow-up period of 17.6 months demonstrated that the median duration of treatment was 8.4 months with atezo and 7.0 months with bev. Additionally, these updated findings highlight that 36% of participants who were initiated on atezo + bev received subsequent systemic therapies, mostly tyrosine kinase inhibitors (32%) [18,20]. Nonetheless, there is a dearth of strong evidence for the most appropriate subsequent therapies post-atezo + bev. The American Society of Clinical Oncology guidelines recommend the use of tyrosine kinase inhibitors, including sorafenib, lenvatinib, regorafenib, and cabozantinib [21], whereas the European Society for Medical Oncology guidelines recommend consideration of all currently approved front- and second-line agents [22]. An observational study using the electronic health record-derived Flatiron Health database (*N* = 856), which presents emerging real-world (RW) treatment patterns, suggested that approximately 70% of individuals with unresectable HCC are now treated with atezo + bev in the USA, with approximately 18% receiving second-line therapies, most commonly tyrosine kinase inhibitors [23].

RW evidence continues to be needed to provide insight into the use of atezo + bev and subsequent therapies for the treatment of advanced or unresectable HCC [24,25]. Therefore, to build on the current evidence base and to address existing gaps in the evidence, this study evaluated RW treatment patterns during and after treatment with atezo + bev for HCC.

## 2. Materials and Methods

### 2.1. Study Design and Eligibility Criteria

This retrospective cohort study used the IQVIA Open-Source Medical (Dx) and Pharmacy (LRx) administrative claims databases, which are representative, payor-agnostic databases with linked longitudinal prescription and medical claims in the USA. Both databases record individual encounter data from participating providers, such as physicians and pharmacies. These databases receive data from all payors, including those with commercial, Medicare, and Medicaid healthcare coverage. Medicare and Medicaid are federal and state government programs that provide healthcare coverage to eligible individuals, such as those ≥65 years of age or those with a low income, respectively. The data were linked through common de-identified patient tokens in both domains of the database. Since enrollment and death information were not available, patient activity in terms of episodes of care was used as a proxy for continuous eligibility and patients were censored on the date of their last observation. The LRx claims were adjudicated, whereas the Dx contained only pre-adjudicated claims.

The study population comprised individuals ≥18 years of age with a diagnosis of HCC who were initiated on atezo + bev between 1 June 2020 and 30 June 2022 (index period). HCC diagnosis was defined as at least one non-ancillary claim with International Classification of Disease, 10th Edition, Clinical Modification [ICD-10-CM] code C22, in any position in all available patient data prior to atezo + bev initiation (index date). Individuals were excluded from the study if they had a diagnosis of any other primary cancer prior to the index date. Eligible individuals were required to have data available for ≥3 months prior to the index date (pre-index period) and for ≥2 months after the index date (post-index period), and to have not received systemic HCC therapy prior to atezo + bev initiation.

Atezo + bev initiation was defined as ≥1 claim for atezo (Healthcare Common Procedure Coding System [HCPCS] J9022 or C9483; National Drug Code [NDC] 50242–917) and ≥1 claim for bev (HCPCS J9035; NDC 50242–060 or 50242–061) on the same day during the index period. To avoid excluding individuals who were initiated on atezo + bev but had claims for the two agents on separate days, a sensitivity analysis was conducted to identify individuals with any claim for bev within the window of ≤1 week prior to and ≤8 weeks after the first observed claim for atezo in the index period; this sensitivity analysis did not identify any additional eligible individuals.

### 2.2. Endpoints and Assessments

**Figure 1:**
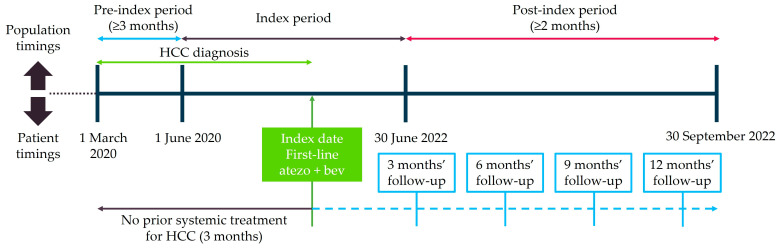
Study design. HCC diagnosis was required any time prior to the date of atezo + bev initiation (index date). Abbreviations: atezo + bev, atezolizumab plus bevacizumab; HCC, hepatocellular carcinoma.

Individuals were followed from the index date until the earliest date of last observation or the end of the study period (30 September 2022; Figure 1). The study endpoints were (1) the proportion of individuals discontinuing atezo + bev and time to discontinuation (TTD), and (2) the proportion of individuals receiving subsequent systemic HCC therapy after atezo + bev and time to subsequent treatment after atezo + bev (time to next treatment [TTNT]). To account for differential follow-up, endpoints were reported in individuals with at least 3, 6, 9, and 12 months of follow-up and over the entire available post-index period.

Subsequent systemic HCC therapies included targeted therapy, immunotherapy, and chemotherapy. Targeted therapy included sorafenib, regorafenib, lenvatinib, cabozantinib, and ramucirumab. Immunotherapy included nivolumab, pembrolizumab, ipilimumab, and nivolumab plus ipilimumab (nivo + ipi) combination therapy. Nivo + ipi was defined as a claim for nivolumab and a claim for ipilimumab on the same date. Individuals who switched to subsequent new therapies (switchers) could still receive atezo + bev concomitantly given that, by definition, claims for any non-atezo + bev therapy in the post-index period were sufficient for the patient to be labeled as a “switcher”.

Discontinuation was defined as a gap of >60 days after the last day of treatment, which was estimated as 21 days after the date of the last claim for the specific agent. Combination therapies (e.g., atezo + bev) were considered as discontinued if both agents were discontinued and the final day of treatment was 21 days after the date of the claim for the last administered agent in the combination. Restarting either atezo or bev after discontinuation was considered the same treatment, to allow for cases of temporary treatment interruptions due to possible intolerance or adverse events.

TTD was defined as the time from the index date to the date of atezo + bev discontinuation and was calculated both for all patients and for only those who discontinued atezo + bev. TTNT was defined as time from the index date to the first post-atezo + bev systemic therapy initiation date.

### 2.3. Statistical Analyses

Descriptive statistics were calculated for all study measures within the overall sample and reported for the entire available post-index period as well as at landmarks at the end of months 3, 6, 9, and 12 after index. Kaplan–Meier (KM) curves were generated for TTD and TTNT, with individuals censored at the end of data availability or end of study period, whichever came first. Death information was not available in the IQVIA Dx and LRx claims databases.

Differences in baseline demographics, clinical characteristics, and outcomes between switchers and individuals not switching to subsequent HCC therapies after atezo + bev (non-switchers) were evaluated using the Chi-square test or Fisher’s test, as appropriate, for categorical variables, and the *t*-test and Wilcoxon rank-sum tests for parametric (mean) and non-parametric (median) measures, respectively. A *p*-value of <0.05 was considered statistically significant. All statistical analyses were conducted using Statistical Analysis System (SAS^®^; Version 9.3 [SAS Institute Inc., Cary, NC, USA]) software.

### 2.4. Ethics

This analysis of the IQVIA Open-Source Dx and LRx administrative claims databases was a secondary data analysis based on patient healthcare information from a de-identified database, from which patients could not be deanonymized. As dictated by Title 45 Code of Federal Regulations (45 CFR 46.101(b)(4)) (available at https://www.govinfo.gov/content/pkg/CFR-2011-title45-vol1/pdf/CFR-2011-title45-vol1.pdf, accessed on 16 November 2023), this analysis was conducted under an exemption from institutional review board oversight for US-based studies using de-identified healthcare records. The research reported in this paper adhered to guidelines set forth by the Helsinki Declaration as revised in 2013.

## 3. Results

### 3.1. Study Population

Of 2515 individuals with a diagnosis of HCC who were initiated on atezo + bev during the study index period (Supplementary Table S1), 825 met the criteria for inclusion in the final study population. Most individuals were ≥65 years of age (64%; median age, 67 years), were male (80%), and had commercial insurance for the index atezo + bev claim (57%). The majority of individuals had a Charlson Comorbidity Index (CCI) score of ≥3 (83%), with the most common comorbidities being hypertension (46%) and diabetes mellitus (30%; Table 1). Most individuals had compensated liver disease. Cirrhosis was present in 51% of individuals, and ascites and hepatic encephalopathy were recorded in 25% and 9% of individuals, respectively; diuretics and lactulose use were recorded in 28% and 9% of individuals, respectively. During the baseline period, esophagogastroduodenoscopy (EGD) was performed in 24% of the study population. Esophageal varices were present in 18% of individuals, and 2% had a prior gastrointestinal hemorrhage. Local therapies—transarterial radioembolization (TARE)/yttrium-90/TARE and transarterial chemoembolization (TACE)—had been previously performed in 12% and 4%, respectively. Distant metastases were present in 22% of individuals at atezo + bev initiation. EGD after atezo + bev initiation was performed in a minority of individuals (4% within 1 month of the index date and 18% more than 1 month after the index date). The median follow-up duration was 15.3 months.

### 3.2. Discontinuation of Atezo + Bev

**Figure 2:**
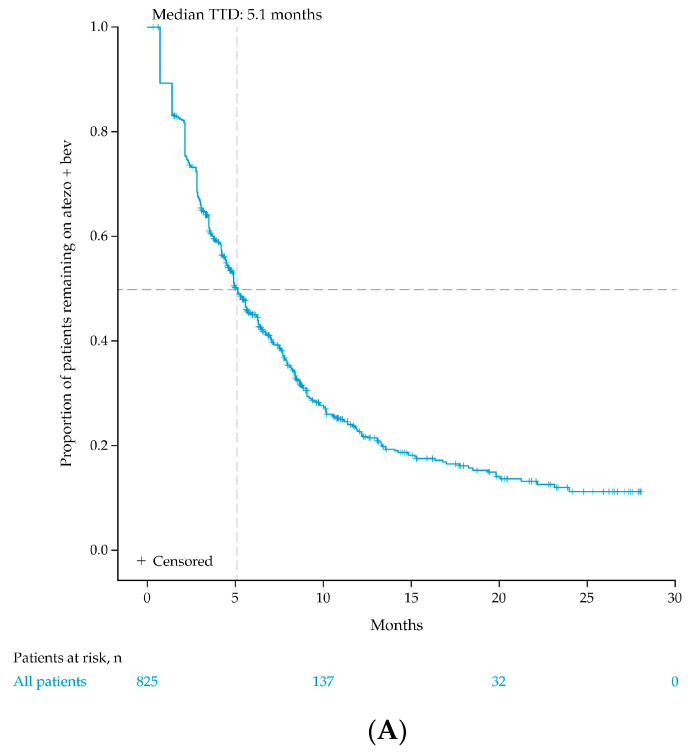

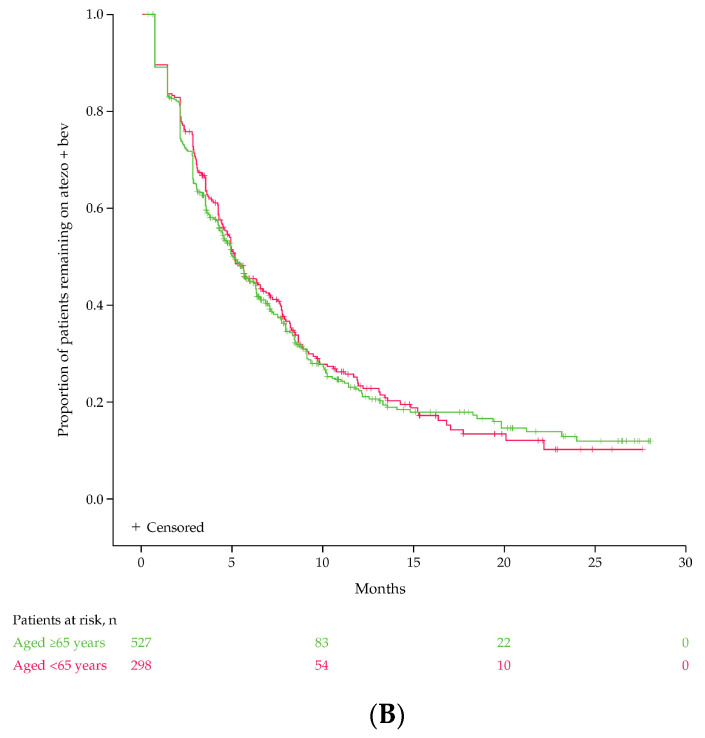
Time to discontinuation in (**A**) all individuals and (**B**) individuals ≥65 years of age over the entire post-index period. Post-index period is defined as the ≥2 months after atezo + bev initiation (index date). Individuals were censored at the end of data availability or the end of study period, whichever came first. Abbreviations: atezo + bev, atezolizumab plus bevacizumab; TTD, time to discontinuation.

Atezo + bev use was discontinued by 72% of individuals over the post-index period (Table 2). The KM-estimated median TTD was 5.1 months for the entire study population (Figure 2), while the calculated median (interquartile range [IQR]) TTD in individuals who discontinued atezo + bev was 3.5 (2.1, 6.3) months (Table 2). In those with at least 3, 6, 9, and 12 months of follow-up, 10%, 43%, 63%, and 77% discontinued atezo + bev, and the median (IQR) TTDs were 0.7 (0.7, 0.7) months, 2.1 (1.4, 2.8) months, 2.8 (1.4, 4.5) months, and 3.5 (2.1, 5.6) months, respectively.

### 3.3. Subsequent Treatments and Treatment Patterns (Sequence) following Atezo + Bev

Overall, a minority of individuals (19%) switched to subsequent systemic HCC therapies. Of those with at least 3, 6, 9, and 12 months of follow-up, 4%, 11%, 16%, and 18% were switchers, respectively (Table 2). Among switchers, the median (IQR) TTNT was 5.4 (3.1, 9.8) months overall, and 2.2 (1.9, 2.5) months, 3.5 (2.3, 4.8) months, 4.4 (2.8, 6.5) months, and 4.9 (3.0, 7.1) months in those with at least 3, 6, 9, and 12 months of follow-up, respectively (Table 2, Figure S1).

Targeted therapy was the most common second and third treatment option, received by 12% and 2% of the study population, respectively, followed by immunotherapy in 4% and 2%, respectively, then chemotherapy in ≤2% (Supplementary Table S2). The multikinase inhibitors lenvatinib (6%) and cabozantinib (4%), and the immunotherapy drug nivolumab (4%), were the most common systemic therapies to which individuals switched following atezo + bev. In individuals with ≥12 months of follow-up, a small proportion received a third and fourth subsequent treatment (4% and 1%, respectively; Supplementary Table S2). Locoregional therapies such as TACE were received by 2% of individuals in the post-index period (Supplementary Table S2).

The non-switchers included 217 individuals who remained on atezo + bev at the end of follow-up and 449 individuals who had discontinued atezo + bev without second-line treatment. Non-switchers who discontinued atezo + bev without second-line therapy were more likely to have a longer median follow-up duration in the post-index period (17.4 months vs. 7.0 months, *p* < 0.001) and to have liver cirrhosis (55% vs. 43%, *p* = 0.005). In individuals with ≥12 months of follow-up, non-switchers who discontinued atezo + bev were also more likely to have comorbid chronic kidney disease (5% vs. 0%, *p* = 0.02), a higher mean CCI score (5.1 vs. 4.6, *p* = 0.04), and prior use of rifaximin (6% vs. 1%, *p* = 0.02; Supplementary Table S3).

The baseline demographics and clinical characteristics, including geographical region, type of insurance claim, sex, comorbidities of interest, and prior medication use, were generally similar for switchers and non-switchers (Table 1). Compared with switchers, non-switchers were more likely to be ≥65 years of age (65% vs. 57%, *p* < 0.03) and have a shorter median follow-up duration in the post-index period (14 months vs. 19 months, *p* < 0.001), and were less likely to have distant metastases (20% vs. 28%, *p* < 0.03; Table 1).

## 4. Discussion

The findings of this RW cohort study demonstrate that 72% of individuals with HCC discontinued atezo + bev within 12 months of initiation, with approximately 19% of individuals initiating subsequent systemic therapies. The most common subsequent therapies amongst switchers (individuals who switched to or added on subsequent new therapies after atezo + bev) were the multikinase inhibitors lenvatinib and cabozantinib and the immunotherapy drug nivolumab.

Similar discontinuation rates and a similar median TTD were demonstrated in a multicenter, RW study in patients with advanced HCC (*N* = 216) who were treated with front-line atezo + bev, in which 66% of the population discontinued treatment with a median TTD of 3.5 months, compared with the 72% discontinuation and observed median TTD of 5.1 months (based on a KM estimate in all individuals) in the present study. In addition, our findings are comparable to another RW study that used the Flatiron Health electronic health record-derived database, which reported a median TTD of 4.8 months and a similar proportion of patients who switched to subsequent therapies (17% vs. 19% in this study) [23].

Notably however, in the IMbrave150 RCT, the duration of treatment was 7.4 months [18]. The shorter duration of atezo + bev treatment in RW settings compared with this RCT may be attributable to differences in treatment settings and protocols, provider expertise, and the study population [25,26]. Moreover, in clinical trials, radiographic progression is often assessed by expert radiologists using strict Response Evaluation Criteria in Solid Tumors (RECIST) specifications, and patients may be taken off therapy earlier in clinical practice due to radiographic progression that does not meet the 20% threshold for progression in RECIST. Other factors, such as the availability of multidisciplinary care and the volume of patients with HCC at selected hospitals, can also differ between clinical trials and RW settings [27,28]. RW populations tend to have higher comorbidities, a worse performance status, and greater liver dysfunction than the well-selected patient populations in clinical trials [29,30,31]. Finally, patients with greater liver dysfunction and performance status have higher competing risks of mortality and often have a shorter time on therapy. Therefore, in extending the findings from existing RCTs, such as IMbrave150 [18] and RW studies [23,32], this study provides further insight into atezo + bev use in RW clinical practice, which includes patients who may be under-represented in RCTs. Nevertheless, further research is encouraged to provide conclusive evidence for the observed differences between RW studies and RCTs.

In this study, multikinase inhibitors were the most common subsequent systemic therapies received by individuals who were first initiated on atezo + bev. This reflects current treatment guidelines, which recommend these agents as suitable treatment options following atezo + bev [21], and research demonstrating the potential efficacy of multikinase inhibitors [33]. In accordance with prior research that suggests that overextending front-line therapy contributes to a deterioration in liver function and overall health status, therefore rendering patients ineligible for second-line therapy [34], the efficacy and safety of multikinase inhibitors in the second-line treatment setting for unresectable HCC warrants further investigation. Thus, research efforts are needed to evaluate the effectiveness of atezo + bev as a front-line therapy in those with advanced HCC who are deemed to be high risk, in order to define upfront those patients who are less likely to benefit from front-line atezo + bev, and to decipher a timely switch to appropriate second- and later-line therapies.

In this study, nearly one-quarter (24%) of individuals underwent EGD prior to atezo + bev initiation, which is lower in comparison with the as-beforementioned multicenter, RW study in patients with advanced HCC (*N* = 216) [32], where 53% of the study population reportedly underwent prior EGD. This could possibly be explained by a difference in the proportion of patients at a high bleeding risk. In the present study, 51% of the population was recorded as having cirrhosis and 18% had portal hypertension, whereas, in the RW study, 80% had cirrhosis. Alternatively, this could reflect regional differences in practice patterns between the USA and Europe, including differential involvement of hepatology services in the clinical care of patients with advanced-stage HCC. Another possible explanation is that data on pre-treatment EGD in this study were collected for only 3 months before atezo + bev initiation. Therefore, further research in assessing the use of pre-treatment EGD over a longer pre-index period and its correlation with treatment-related bleeding events is recommended.

Limitations of this study are acknowledged. First, open-source claims data are collected primarily for reimbursement; therefore, the data may not completely describe the clinical diagnoses, treatment, and disease progression. Second, the findings of this study may be subject to selection bias as the use of the newly approved atezo + bev combination therapy may depend on the treating clinicians’ awareness of the relevant drug approvals. Third, death records were not captured and the expected number of individuals switching to subsequent systemic therapies and the duration of follow-up are limited. Fourth, liver-related decompensation and relevant medications were used as indicators of liver dysfunction; however, Child–Pugh and albumin–bilirubin scores were not available. Therefore, conventional measures of liver function could not be used. In addition, data on Eastern Cooperative Oncology Group performance status, Barcelona Clinic Liver Cancer stage, and vascular invasion are not captured in claims data. For this reason, it could not be evaluated whether discontinuation and switch differed in individuals in this study who were similar to those in the atezo + bev cohort in the IMbrave150 RCT. Finally, this study does not report reasons for discontinuation or switches to subsequent therapies because this information is not captured in claims data. Nevertheless, treatment discontinuation is known to be frequent in patients with advanced HCC, owing to disease progression or significant toxicity [35,36], which could be explored in future research.

## 5. Conclusions

In conclusion, RW treatment patterns in this study demonstrated that most individuals diagnosed with HCC discontinued atezo + bev combination therapy within 12 months of initiation, and a minority switched to subsequent systemic HCC therapies after atezo + bev discontinuation. Further research is required to explore reasons for these treatment patterns, provide insight into the effectiveness of atezo + bev in RW patient populations, and to decipher a timely switch to appropriate subsequent systemic therapies. Research efforts are also required to define upfront those patients who are unlikely to benefit from atezo + bev and identify optimal front-line treatment options for this subgroup.

## Figures and Tables

**Table 1:** Baseline demographics and disease characteristics.

Characteristics	Overall (*N* = 825)	Switchers * (*n* = 159)	Non-Switchers ^†^ (*n* = 666)
Age, years			
Mean (SD)	67 (9)	65 (10)	67 (9)
Median	67	66	67
Age group, years, *n* (%)			
18–34	8 (1)	3 (2)	5 (1)
35–44	11 (1)	4 (3)	7 (1)
45–54	34 (4)	12 (8)	22 (3)
55–64	245 (30)	49 (31)	196 (29)
≥65	527 (64)	91 (57)	436 (65)
Male sex, *n* (%)	663 (80)	135 (85)	528 (79)
Geographical region, *n* (%)			
South	319 (39)	66 (42)	253 (38)
West	228 (28)	30 (19)	198 (30)
Midwest	169 (20)	38 (24)	131 (20)
Northeast	108 (13)	25 (16)	83 (12)
Unknown	1 (<1)	0 (0)	1 (<1)
Insurance type on index claim, *n* (%)			
Commercial	470 (57)	97 (61)	373 (56)
Medicare	324 (39)	58 (36)	266 (40)
Medicaid	17 (2)	3 (2)	14 (2)
Unknown	14 (2)	1 (1)	13 (2)
Liver disease etiology ^‡^, *n* (%)			
Hepatitis C	166 (20)	33 (21)	133 (20)
Alcohol abuse	45 (5)	11 (7)	34 (5)
Hepatitis B	37 (4)	9 (6)	28 (4)
Liver-related comorbidities ^‡^, *n* (%)			
Cirrhosis	423 (51)	82 (52)	341 (51)
Ascites ^§^	204 (25)	36 (23)	168 (25)
Esophageal varices	148 (18)	23 (14)	125 (19)
Hepatic encephalopathy ^¶^	73 (9)	14 (9)	59 (9)
Gastrointestinal hemorrhage	14 (2)	4 (3)	10 (2)
Portal hypertension	149 (18)	23 (14)	126 (19)
Other comorbidities of interest, *n* (%)			
Hypertension	381 (46)	71 (45)	310 (47)
Diabetes (type 2)	242 (29)	42 (26)	200 (30)
Heart failure	37 (4)	6 (4)	31 (5)
Chronic kidney disease	34 (4)	10 (6)	24 (4)
Myocardial infarction	8 (1)	1 (1)	7 (1)
Cerebral hemorrhage (stroke)	5 (1)	1 (1)	4 (1)
Diabetes (type 1)	2 (<1)	1 (1)	1 (<1)
CCI (Dartmouth–Manitoba adaptation) Mean (SD)	5.0 (2.5)	5.2 (2.6)	4.9 (2.5)
CCI category, *n* (%)			
0	9 (1)	2 (1)	7 (1)
1	4 (<1)	0 (0)	4 (1)
2	130 (16)	19 (12)	111 (17)
≥3	682 (83)	138 (87)	544 (82)
Other medications, *n* (%)			
Analgesics	499 (60)	99 (62)	400 (60)
Antihypertensives **	464 (56)	80 (50)	384 (58)
Diuretics	231 (28)	39 (25)	192 (29)
Antithrombotic agents	175 (21)	32 (20)	143 (21)
Systemic corticosteroids	136 (16)	31 (19)	105 (16)
Lactulose	78 (9)	14 (9)	64 (10)
Rifaximin	34 (4)	6 (4)	28 (4)
Prior EGD, *n* (%)	199 (24)	42 (26)	157 (24)
Metastases present, *n* (%)	180 (22)	45 (28)	135 (20)
Prior procedures, *n* (%)			
TARE/Y90/TARE	97 (12)	20 (13)	77 (12)
TACE ^††^	32 (4)	5 (3)	27 (4)
Radiation therapy	29 (4)	7 (4)	22 (3)
Ablation	8 (1)	4 (3)	4 (1)
Resection/partial hepatectomy	3 (<1)	1 (1)	2 (<1)
Median follow-up time ^‡‡^, months	15.3	19.2	14.3

* Switchers are defined as individuals with any claims in addition to atezo + bev in the post-index period; ^†^ non-switchers are defined as individuals with no claims other than atezo + bev in the post-index period; ^‡^ multiple responses; ^§^ ascites definition includes any of the following: any ascites ICD code, any cirrhosis ICD code (alongside loop diuretics and potassium-sparing diuretics), or paracentesis/thoracentesis procedure code; ^¶^ encephalopathy definition includes any of the following: any encephalopathy ICD code, prescription for lactulose, or prescription for rifaximin; ** antihypertensives include medications that treat hypertension, including those used in combination with diuretics; ^††^ any of the following chemotherapies are considered to be TACE if two or fewer claims for the same therapy are observed in the 3-month pre-index period: doxorubicin, doxorubicin HCl cisplatin, doxorubicin HCl liposome, epirubicin, epirubicin HCl, mitomycin, mitoxantrone HCl, gemcitabine HCl, and idarubicin HCl; ^‡‡^ follow-up time is defined as time from index to minimum of last LRx claim, last Dx claim, last pharmacy stability date, and last provider stability date. Abbreviations: atezo + bev, atezolizumab plus bevacizumab; CCI, Charlson Comorbidity Index; EGD, esophagogastroduodenoscopy; HCl, hydrochloride; ICD, International Classification of Diseases; SD, standard deviation; TACE, transarterial chemoembolization; TARE, transarterial radioembolization; Y90, yttrium-90.

**Table 2:** Discontinuation of atezo + bev and switching patterns.

Outcomes	Duration of Follow-Up				
	Overall (*N* = 825)	≥3 Months (*n* = 749)	≥6 Months (*n* = 711)	≥9 Months (*n* = 623)	≥12 Months (*n* = 548)
Discontinuation of atezo + bev *, *n* (%)	593 (72)	76 (10)	309 (43)	393 (63)	421 (77)
Time to discontinuation *, months					
Mean (SD)	4.6 (4.0)	0.7 (0)	2.1 (1.0)	3.1 (1.8)	3.9 (2.5)
Median (IQR)	3.5 (2.1, 6.3)	0.7 (0.7, 0.7)	2.1 (1.4, 2.8)	2.8 (1.4, 4.5)	3.5 (2.1, 5.6)
No discontinuation of atezo + bev, *n* (%)	232 (28)	673 (90)	402 (57)	230 (37)	127 (23)
Switchers ^†^, any treatment, *n* (%)	159 (19)	32 (4)	76 (11)	100 (16)	100 (18)
Targeted therapy	104 (13)	20 (3)	51 (7)	69 (11)	64 (12)
Immunotherapy	42 (5)	5 (1)	16 (2)	21 (3)	29 (5)
Chemotherapy	29 (4)	7 (1)	13 (2)	14 (2)	17 (3)
Time to next treatment, months					
Mean (SD)	7.5 (6.0)	2.1 (0.7)	3.5 (1.4)	4.6 (2.2)	5.1 (2.7)
Median (IQR)	5.4 (3.1, 9.8)	2.2 (1.9, 2.5)	3.5 (2.3, 4.8)	4.4 (2.8, 6.5)	4.9 (3.0, 7.1)
Non-switchers ^‡^, *n* (%)	666 (81)	717 (96)	635 (89)	523 (84)	448 (82)
Yes	217 (26)	–	380 (53)	–	121 (22)
No	449 (54)	–	255 (36)	–	327 (60)

* Discontinuation of atezo + bev is defined as a gap of >21 plus 60 days after the last atezo or bev administration. Atezo + bev combination therapy is considered to be discontinued if both agents are discontinued. Atezo or bev monotherapy counts as ongoing treatment. TTD is defined as time from index date to date of last administration of atezo or bev plus 21 days; ^†^ switchers are defined as individuals with any claims in addition to atezo + bev in the post-index period; ^‡^ non-switchers are defined as individuals with no claims other than atezo + bev. Individuals who restarted atezo + bev after discontinuation are also considered non-switchers. Abbreviations: atezo + bev, atezolizumab plus bevacizumab; IQR, interquartile range; SD, standard deviation; TTD, time to discontinuation.

## Data Availability

The data that support the findings of this study are available from IQVIA, which were used under license for the current study, and so are not publicly available. Interested researchers should contact IQVIA (https://www.iqvia.com/solutions/real-world-evidence/real-world-data-and-insights, accessed on 16 November 2023).

## Supplementary Materials

The following supporting information can be downloaded at: https://www.mdpi.com/article/10.3390/cancers15235532/s1, Figure S1: Time to next treatment among all individuals over the entire post-index period; Table S1: Attrition of the study sample of (a) all individuals receiving front-line atezolizumab plus bevacizumab and (b) individuals receiving front-line atezolizumab plus bevacizumab with initiation on different days; Table S2: Treatment distribution by sequence in overall study population and individuals with at least 3, 6, 9, and 12 months of follow-up; Table S3: Baseline demographics and disease characteristics for non-switchers who did and did not remain on both atezolizumab plus bevacizumab at different time points of follow-up.
