# Supplementary material for: Real-World Systemic Treatment Patterns after Atezolizumab and Bevacizumab in Patients with Hepatocellular Carcinoma in the United States
Source: Cancers (Basel). 2023 Nov 22;15(23):5532. doi: 10.3390/cancers15235532 (PMC10705135; doi:10.3390/cancers15235532)
Supplement: Supplementary file 1 [file cancers-15-05532-s001.zip › cancers-2699750-supplementary.pdf]

cancers

Supplementary Materials

Real-World Systemic Treatment Patterns after Atezolizumab and Bevacizumab in Patients with Hepatocellular Carcinoma in the United States

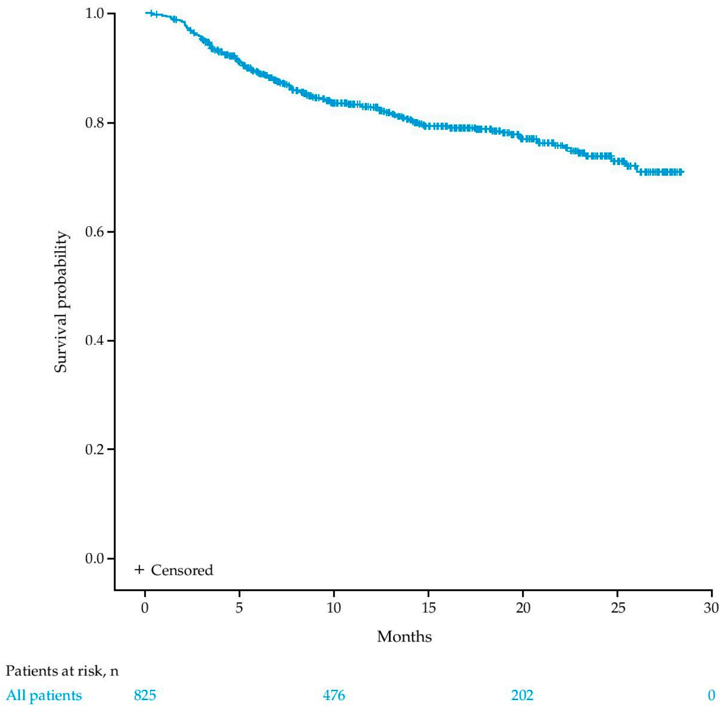

Supplementary Figure S1. Time to next treatment among all individuals over the entire post-index period. Post-index period defined as the ≥2 months after atezo+bev initiation (index date). Individuals were censored at the end of data availability or end of the study period, whichever came first. Switchers defined as individuals with claims for any therapy other than atezo+bev in the post-index period. Abbreviations: atezo+bev, atezolizumab plus bevacizumab.

Supplementary Table S1. Attrition of the study sample of (**a**) all individuals receiving front-line atezolizumab plus bevacizumab and (**b**) individuals receiving front-line atezolizumab plus bevacizumab with initiation on different days

(**a**)

Steps	Criteria	Excluded, *n* (%)	Remaining, *n* (%)
1	Individuals with ≥1 claim with an HCC diagnosis code * in any position in all available data prior to the index date (date of initiation of combination therapy)	758 (13)	2,515 (44)
2	Individuals without any systemic therapy (sorafenib, regorafenib, lenvatinib, cabozantinib, nivolumab, pembrolizumab, ramucirumab, and ipilimumab) in the 3-month pre-index period	85 (1)	2,430 (42)
3	Individuals without any non-TACE † chemotherapy of interest in the 3-month pre-index period	38 (1)	2,392 (42)
4	Individuals ≥18 years of age on the index date	4 (<1)	2,388 (42)
5	Individuals with ≥1 pharmacy claim in LRx and ≥1 medical claim in Dx in the 3-month pre-index period	194 (3)	2,194 (38)
6	Individuals with pharmacy and provider stability ‡ for the 3-month pre-index period	319 (6)	1,875 (33)
7	Individuals with ≥1 pharmacy claim in LRx and ≥1 medical claim in Dx in the 2-month post-index period	435 (8)	1,440 (25)
8	Individuals with pharmacy and provider stability ‡ for the 2-month post-index period	10 (<1)	1,430 (25)
9	Exclusion of individuals with ≥1 diagnosis code for any primary cancer types in all available data prior to the index date (including the index date)	605 (11)	825 (14)

(**b**)

Steps	Criteria	Excluded, *n* (%)	Remaining, *n* (%)
1	Individuals with ≥1 claim with an HCC diagnosis code * in any position in all available data prior to the index date (latter claim for atezo or bev)	28 (1)	13 (1)
2	Individuals without any systemic therapy (sorafenib, regorafenib, lenvatinib, cabozantinib, nivolumab, pembrolizumab, ramucirumab, and ipilimumab) in the 3-month pre-index period	2 (<1)	11 (<1)
3	Individuals without any non-TACE † chemotherapy of interest in the 3-month pre-index period	0 (0)	11 (<1)
4	Individuals ≥18 years of age on the index date	0 (0)	11 (<1)
5	Individuals with ≥1 pharmacy claim in LRx and ≥1 medical claim in Dx in the 3-month pre-index period	0 (0)	11 (<1)
6	Individuals with pharmacy and provider stability ‡ for the 3-month pre-index period	1 (<1)	10 (<1)
7	Individuals with ≥1 pharmacy claim in LRx and ≥1 medical claim in Dx in the 2-month post-index period	2 (<1)	8 (<1)
8	Individuals with pharmacy and provider stability ‡ for the 2-month post-index period	8 (<1)	0 (0)

9	Exclusion of individuals with ≥1 diagnosis code for any primary cancer types in all available data prior to the index date (including the index date)	–	–

* HCC diagnosis code (ICD-10: C22.*; ICD-9: 155.*); † Any of the following chemotherapies are considered to be TACE if ≤2 claims for the same therapy are observed in the 3-month pre-index period: doxorubicin, doxorubicin HCl cisplatin, doxorubicin HCl liposome, epirubicin, epirubicin HCl, mitomycin, mitoxantrone HCl, gemcitabine HCl, and idarubicin HCl; ‡ Any pharmacy/provider the individual visited during the 12-month pre-index period and anytime post index. Abbreviations: atezo, atezolizumab; bev, bevacizumab; Dx, IQVIA medical claims data; HCC, hepatocellular carcinoma; HCl, hydrochloride; ICD, International Classification of Disease; LRx, IQVIA longitudinal prescription data; TACE, transarterial chemoembolization.

Supplementary Table S2. Treatment distribution by sequence in overall study population and individuals with at least 3, 6, 9, and 12 months of follow-up

**Treatments, *n* (%)**	**Duration of follow-up**				
	**Overall (*N* = 825)**	**≥3 months (*n* = 749)**	**≥6 months (*n* = 711)**	**≥9 months (*n* = 623)**	**≥12 months (*n* = 548)**
**First treatment**					
Atezo+bev	825 (100)	749 (100)	711 (100)	623 (100)	548 (100)
Discontinued first treatment without second treatment at last follow-up	456 (55)	72 (10)	256 (36)	308 (49)	330 (60)
Remaining on first treatment at last follow-up	221 (27)	648 (87)	384 (54)	219 (35)	123 (22)
**Second treatment, any**	148 (18)	29 (4)	71 (10)	96 (15)	95 (17)
Targeted therapy	99 (12)	20 (3)	51 (7)	69 (11)	63 (11)
Immunotherapy	36 (4)	5 (1)	15 (2)	19 (3)	24 (4)
Chemotherapy *	13 (2)	4 (1)	5 (1)	8 (1)	8 (1)
Discontinued second treatment without third treatment at last follow-up	80 (10)	0	8 (1)	35 (6)	49 (9)
Remaining on second treatment at last follow-up	38 (5)	26 (3)	54 (8)	50 (8)	28 (5)
**Third treatment, any**	30 (4)	3 (<1)	9 (1)	11 (2)	18 (3)
Targeted therapy	14 (2)	0	3 (<1)	5 (1)	8 (1)
Immunotherapy	6 (1)	0	0	1 (<1)	4 (1)
Chemotherapy *	10 (1)	3 (<1)	6 (1)	5 (1)	6 (1)
Discontinued third treatment without fourth treatment at last follow-up	11 (1)	0	2 (<1)	4 (1)	4 (1)
Remaining on third treatment at last follow-up	10 (1)	3 (<1)	4 (1)	5 (1)	10 (2)
**Fourth treatment, any**	9 (1)	0	3 (<1)	2 (<1)	4 (1)
Targeted therapy	2 (<1)	0	0	0	0
Immunotherapy	2 (<1)	0	1 (<1)	1 (<1)	2 (<1)
Chemotherapy *	5 (1)	0	2 (<1)	1 (<1)	2 (<1)
**Post-index TACE †**	32 (4)	6 (1)	10 (1)	8 (1)	14 (3)
**EGD within 1 month post index**	37 (4)	33 (4)	32 (5)	29 (5)	25 (5)
**EGD >1 month post index**	151 (18)	143 (19)	138 (19)	131 (21)	121 (22)

* Two consecutive claims for non-TACE chemotherapy. † Post-index TACE defined as NDC and HCPCS for any chemotherapy used for TACE (doxorubicin, doxorubicin HCl, cisplatin, doxorubicin HCl liposome, epirubicin, epirubicin HCl, mitomycin, and mitoxantrone HCl) in the post-index period, regardless of whether a TACE procedure code ±3 days from the TACE chemotherapy is or is not observed in the post-index period. Abbreviations: atezo+bev, atezolizumab plus bevacizumab; EGD, esophagogastroduodenoscopy; HCl, hydrochloride; HCPCS, Healthcare Common Procedure Coding System; NDC, National Drug Code; TACE, transarterial chemoembolization.

Supplementary Table S3. Baseline demographics and disease characteristics for non-switchers who did and did not remain on both atezolizumab plus bevacizumab at different time points of follow-up

**Characteristics**	**Duration of follow-up**								
	**Overall**			**≥6 months**			**≥12 months**		
	**Non-Switchers (*N* = 825)**			**Non-Switchers (*n* = 711)**			**Non-Switchers (*n* = 548)**		
	**Remained on atezo+bev ***			**Remained on atezo+bev ***			**Remained on atezo+bev ***		
	**Yes (*n* = 217)**	**No (*n* = 449)**	**P-value**	**Yes (*n* = 380)**	**No (*n* = 255)**	**P-value**	**Yes (*n* = 121)**	**No (*n* = 327)**	**P-value**
Age, years									
Mean (SD)	68 (10)	67 (9)	0.759	67 (8)	67 (10)	0.848	67 (7)	67 (9)	0.964
Median	68	67	0.398	67	67	0.773	66	67	0.833
Age group, years, *n* (%)			0.629			0.059			0.484
18–34	2 (1)	3 (1)		0	5 (2)		0	5 (2)	
35–44	2 (1)	5 (1)		5 (1)	2 (1)		0	2 (1)	
45–54	10 (5)	12 (3)		15 (4)	6 (2)		6 (5)	10 (3)	
55–64	59 (27)	137 (31)		121 (32)	78 (31)		43 (36)	104 (32)	
≥65	144 (66)	292 (65)		239 (63)	164 (64)		72 (60)	206 (63)	
Male sex, *n* (%)	174 (80)	354 (79)	0.760	308 (81)	197 (77)	0.245	100 (83)	252 (77)	0.201
Geographical region, *n* (%)			0.733			0.261			0.214
South	84 (39)	169 (38)		145 (38)	101 (40)		43 (36)	121 (37)	
West	62 (29)	136 (30)		106 (28)	78 (31)		34 (28)	100 (31)	
Midwest	44 (20)	87 (19)		88 (23)	43 (17)		33 (27)	62 (19)	
Northeast	26 (12)	57 (13)		41 (11)	33 (13)		11 (9)	44 (13)	
Unknown	1 (<1)	0		0	0		0	0	
Insurance type on index claim, *n* (%)			0.573			0.780			0.129

Commercial	121 (56)	252 (56)		220 (58)	141 (55)		69 (57)	188 (57)	
Medicare	85 (39)	181 (40)		144 (38)	105 (41)		46 (38)	129 (39)	
Medicaid	7 (3)	7 (2)		7 (2)	5 (2)		0	5 (2)	
Unknown	4 (2)	9 (2)		9 (2)	4 (2)		6 (5)	5 (2)	
Liver disease etiology †, *n* (%)									
Hepatitis C	39 (18)	94 (21)	0.409	81 (21)	47 (18)	0.374	28 (23)	69 (21)	0.642
Alcohol abuse	13 (6)	21 (5)	0.459	19 (5)	15 (6)	0.628	9 (7)	11 (3)	0.064
Hepatitis B	8 (4)	20 (4)	0.837	10 (3)	12 (5)	0.161	3 (2)	11 (3)	0.768
Liver-related comorbidities †, *n* (%)									
Cirrhosis	94 (43)	247 (55)	0.005	194 (51)	138 (54)	0.448	55 (45)	181 (55)	0.063
Ascites ‡	46 (21)	122 (27)	0.106	87 (23)	70 (27)	0.192	23 (19)	90 (28)	0.065
Esophageal varices	40 (18)	85 (19)	0.916	73 (19)	47 (18)	0.806	23 (19)	62 (19)	0.991
Portal hypertension	35 (16)	91 (20)	0.208	66 (17)	49 (19)	0.554	19 (16)	65 (20)	0.315
Hepatic encephalopathy §	20 (9)	39 (9)	0.884	39 (10)	22 (9)	0.493	12 (10)	27 (8)	0.580
Gastrointestinal hemorrhage	3 (1)	7 (2)	1.000	7 (2)	4 (2)	1.000	1 (1)	6 (2)	0.680
Metastases present, *n* (%)	50 (23)	85 (19)	0.219	75 (20)	53 (21)	0.747	21 (17)	69 (21)	0.380
Other comorbidities of interest, *n* (%)									
Hypertension	109 (50)	201 (45)	0.214	186 (49)	113 (44)	0.252	58 (48)	146 (45)	0.535
Diabetes (type 2)	62 (29)	138 (31)	0.590	114 (30)	81 (32)	0.637	32 (26)	101 (31)	0.361
Heart failure	10 (5)	21 (5)	1.000	19 (5)	15 (6)	0.628	6 (5)	19 (6)	0.727
Chronic kidney disease	5 (2)	19 (4)	0.270	15 (4)	13 (5)	0.489	0	15 (5)	0.015
Myocardial infarction	2 (1)	5 (1)	1.000	4 (1)	3 (1)	1.000	2 (2)	2 (1)	0.296
Cerebral hemorrhage (stroke)	1 (<1)	3 (1)	1.000	1 (<1)	2 (1)	0.568	1 (1)	2 (1)	1.000
Diabetes (type 1)	0	1 (<1)	1.000	1 (<1)	1 (<1)	1.000	1 (1)	0	0.270
CCI (Dartmouth–Manitoba adaptation)									

Mean (SD)	4.9 (2.5)	5.0 (2.5)	0.556	4.9 (2.5)	5.1 (2.5)	0.270	4.6 (2.4)	5.1 (2.6)	0.035
CCI category, *n* (%)			0.050			0.160			0.032
0	1 (<1)	6 (1)		3 (1)	5 (2)		1 (1)	6 (2)	
1	2 (1)	2 (<1)		1 (<1)	2 (1)		1 (1)	0	
2	47 (22)	64 (14)		60 (16)	29 (11)		25 (21)	40 (12)	
≥3	167 (77)	377 (84)		316 (83)	219 (86)		94 (78)	281 (86)	
Prior treatment, *n* (%)									
Antihypertensives ¶	134 (62)	250 (56)	0.155	224 (59)	141 (55)	0.361	69 (57)	185 (57)	0.932
Analgesics	133 (61)	267 (59)	0.674	234 (62)	153 (60)	0.689	68 (56)	200 (61)	0.341
Diuretics	66 (30)	126 (28)	0.525	108 (28)	70 (27)	0.790	30 (25)	90 (28)	0.562
Antithrombotic agents	45 (21)	98 (22)	0.841	83 (22)	55 (22)	0.935	27 (22)	71 (22)	0.891
Systemic corticosteroids	37 (17)	68 (15)	0.571	60 (16)	43 (17)	0.719	19 (16)	55 (17)	0.777
Lactulose	19 (9)	45 (10)	0.675	36 (9)	30 (12)	0.354	11 (9)	33 (10)	0.752
Rifaximin	6 (3)	22 (5)	0.223	18 (5)	10 (4)	0.624	1 (1)	19 (6)	0.023
Antihemorrhagics	0	0	–	0	0	–	0	0	–
Prior EGD, *n* (%)	53 (24)	104 (23)	0.770	95 (25)	51 (20)	0.142	29 (24)	72 (22)	0.661
Prior procedures, *n* (%)									
TARE/Y90/TARE	26 (12)	51 (11)	0.798	48 (13)	28 (11)	0.530	12 (10)	42 (13)	0.398
TACE **	5 (2)	22 (5)	[a]	13 (3)	16 (6)	[a]	3 (2)	20 (6)	[a]
Radiation therapy	6 (3)	16 (4)	0.652	12 (3)	11 (4)	0.445	4 (3)	13 (4)	1.000
Resection/partial hepatectomy	1 (<1)	1 (<1)	0.546	1 (<1)	1 (<1)	1.000	1 (1)	1 (<1)	0.468
Ablation	0	4 (1)	0.310	3 (1)	3 (1)	0.689	0	5 (2)	0.330
Median follow-up time ††, months	7.0	17.4	<0.001	–	–	–	–	–	–

* Non-switchers defined as individuals with no claims other than atezo+bev. Individuals who restarted atezo+bev after discontinuation are also considered non-switchers; † Multiple responses; ‡ Ascites definition includes any of the following: any ascites ICD code, any cirrhosis ICD code (alongside loop diuretics and potassium-sparing diuretics), or paracentesis/thoracentesis procedure code; § Encephalopathy definition includes any of the following: any encephalopathy ICD code, prescription for lactulose, or prescription for rifaximin; ¶ Antihypertensives include medications that treat hypertension including those used in combination with diuretics; ** Any of the following chemotherapy is considered to be TACE if ≤2 claims for the same therapy are observed in the 3-month pre-index period: doxorubicin, doxorubicin HCl cisplatin, doxorubicin HCl liposome, epirubicin,

epirubicin HCl, mitomycin, mitoxantrone HCl, gemcitabine HCl, and idarubicin HCl; [††] Follow-up time defined as time from index to minimum of last LRx claim, last Dx claim, last pharmacy stability date, and last provider stability date; [a] Corresponding p-values for TACE/TACE (drugs only) for both non-switcher groups over entire follow-up, ≥6 months of follow-up, and ≥12 months of follow-up, respectively were $p = 0.512$, $p = 0.128$, and $p = 0.689$/$p = 0.293$, $p = 0.388$, and $p = 0.374$. Abbreviations: atezo+bev, atezolizumab plus bevacizumab; CCI, Charlson Comorbidity Index; Dx, IQVIA medical claims data; EGD, esophagogastroduodenoscopy; HCl, hydrochloride; ICD, International Classification of Disease; LRx, IQVIA longitudinal prescription data; SD, standard deviation; TACE, transarterial chemoembolization; TARE, transarterial radioembolization; Y90, yttrium-90.
